# Parental Cancer: Acceptance and Usability of an Information Booklet for Affected Parents

**DOI:** 10.3389/fpsyg.2022.769298

**Published:** 2022-02-24

**Authors:** Leslie Melchiors, Wiebke Geertz, Laura Inhestern

**Affiliations:** ^1^Department of Medical Psychology, University Medical Center Hamburg-Eppendorf, Hamburg, Germany; ^2^Clinical Psychology, Helmut-Schmidt-University/University of the Federal Armed Forces, Hamburg, Germany

**Keywords:** parental cancer, information needs, information booklet, qualitative evaluation, psychosocial support, acceptance and usability

## Abstract

**Background:**

Parents affected by cancer are confronted with challenges such as communicating with their children about the disease and dealing with changes in their parental role. Providing appropriate information could support affected parents and their children. Still, high-quality and information booklets are rare. Therefore, we developed an information booklet for affected families. The study aims are: (1) investigating the acceptability and usability of the information booklet, (2) determining parental information needs, and (3) collating suggestions for implementation. Finally, we adapted the booklet according to the findings.

**Methods:**

We interviewed five experts in psychosocial care of parental cancer and nine affected parents using a semi-structured interview guideline. Participants received the developed booklet after giving the consent to participate. Interviews on acceptability and usability of the booklet and information needs were conducted about 1 week after receiving the booklet. The interviews were analyzed using structuring content analysis.

**Results:**

(1) Experts and parents reported that the information booklet addresses the experiences of affected families and that the content combination makes it useful in an unprecedented way indicating both acceptance and usability. (2) Following dimensions were identified as information needs: (a) communication, (b) support offers, (c) children’s disease understanding and needs, (d) organization of family life, (e) competence in parenting, and (f) sources of additional information material. (3) The booklet should be handed out personally by, e.g., healthcare professionals and might be accompanied by a personal counseling. Minor adaptations on language and content were conducted based on the findings.

**Conclusion:**

Indicated acceptance and usability of the developed information booklet for parents with cancer suggest a low-threshold, basic support for affected families and health professionals. The diverse parental information needs are covered. The long-term effects of the booklet, e.g., on the use of psychosocial support offers, parental self-efficacy and psychological distress will be quantitatively investigated.

## Introduction

A cancer diagnosis of a parent affects the whole family (Ernst et al., 2010). Cancer patients experience physical and psychological health impairments through the disease itself or its treatment. Additionally, cancer patients parenting minor children can face social and practical changes and challenges with regard to their parental role and their family lives, e.g., regarding maintaining family routines or child-care duties (Inhestern and Bergelt, 2018). Ill parents show a decreased quality of life and impaired mental health in comparison to cancer patients without minor children (Ernst et al., 2010; Park et al., 2016). Compared to a control sample without cancer, parents with cancer report a decreased parental self-efficacy (Cessna et al., 2016). Children affected by parental cancer are exposed to a new living situation and threatened with the potential loss of a parent (Huizinga et al., 2011).

Parents with cancer report that communication about cancer within the family (e.g., about diagnosis, treatment, but also feelings and needs with regard to parental diagnosis) is one of the biggest challenges and that they have a need for additional information and support (Kennedy and Lloyd-Williams, 2009). Many parents would appreciate advice and guidance in this new situation, especially when it comes to talking with their children about cancer and how it affects everyone (Thastum et al., 2008). Also, children affected by parental cancer report high levels of unmet needs with the need for information being the most frequent one (Kennedy and Lloyd-Williams, 2009; Ellis et al., 2017; Ghofrani et al., 2019).

International guidelines on cancer care recommend addressing the psychosocial and information needs of cancer patients as well as the needs of their close relatives (Riba et al., 2019). In Germany, the clinical practice guideline for psycho-oncology claims the provision of psycho-oncological support for patients and relatives (Weis et al., 2014). Nevertheless, psychosocial support for families affected by parental cancer in Germany is scattered with heterogeneous and limited support offers (Ernst et al., 2011). Often, healthcare professionals do not include information about parental issues into routine care due to limited capacities (e.g., time or knowledge; Turner et al., 2007). Therefore, main information sources, besides healthcare professionals, are online resources and booklets (Gaisser et al., 2016). Considering the aim of implementing stepped-care models in psycho-oncological care, self-help materials and written information can provide a low-threshold support in terms of a basic intervention for all cancer patients (Krebber et al., 2012; Lewis et al., 2012). However, the quality and suitability of psycho-oncologic booklets and online materials are essential to meet the needs of cancer patients. A study by Brütting et al. (2019), examining the quality, readability, and comprehensibility of currently available German information brochures for melanoma patients highlights the need for a didactic and quality-related revision of available brochures. The authors emphasize the need for information and brochures to be understandable and readable for patients. An international scoping review on online information resources for cancer patients parenting minor children identified several resources in English language (Weeks et al., 2019). Still, the results underline a need for high quality information and a comprehensive development of information resources for affected parents (Weeks et al., 2019). High quality information material, e.g., should provide clear aims and content according to the aims, should be suited for the readership and provide information about the context and date of publication (Charnock et al., 1999; Weeks et al., 2019). To our knowledge, there is no systematic evaluation of available psycho-oncological information for families affected by parental cancer in German language. Still, based on the clinical impression and expertise a lack of accessible information material including information for parents throughout the course of disease, information on age-specific communication and developmental aspects of children, hands-on suggestions for daily routines for families as well as an overview of local support offers for affected families including contact details was identified. Therefore, we developed an information booklet for parents with cancer parenting minor children addressing these elements.

In this qualitative pilot study, our aims were (1) to investigate the acceptability and feasibility of the developed information booklet, (2) to assess the information needs to adapt the brochure, and (3) to collate suggestions for implementation of the information booklet into routine care. Finally, we adapted the booklet according to our findings.

## Materials and Methods

### Study Design

The study follows a multiperspective qualitative approach to investigate the study aims. Participants (parents with cancer, partners of parents with cancer, and experts in the field of psychosocial care for families affected by parental cancer) were interviewed after receiving a draft version of the developed information booklet for cancer patients parenting minor children. Consecutive sampling strategy was used for the recruitment of participants. After providing written informed consent and receiving the information booklet, an interview appointment was arranged. The study was approved by the local Ethics Committee (Ethics Committee of the Chamber of Psychotherapists of Hamburg, 06/2018-PTK-HH).

### Information Booklet

The study team consisting of psychologists with expertise in (child- and adolescent-) psychotherapy, family-centered counseling and psycho-oncology (LM, LI, and WG) reviewed existing brochures and booklets for affected parents in German as well as relevant homepages. Based on their clinical practice, they developed the first draft of the information booklet, which was extensively revised by experienced psychologists with longtime expertise in psychotherapy, family-centered care, and psycho-oncology (FSK and CB). The study team worked with an illustrator, who designed illustrations. Requirements for the illustrations were to include diverse family constellations and to release from tight gender attributes to enable parents and children to identify. The draft of the booklet was typeset by a typesetter of the University Medical Center Hamburg.

The topics of the booklet were chosen based on the clinical experiences in counseling cancer-affected parents as well as identified relevant themes addressed in other information material. Disagreement on the content and key aspects were debated within the team and resolved based on research or by majority vote. Where applicable, we oriented to the DISCERN-Checklist, an instrument for judging the quality of written consumer health information on treatment choices (Charnock et al., 1999). As our aim was to provide the information in an understandable and readable manner, we included illustrations and supporting elements, such as text boxes highlighting parts of the content or recommendations for daily routines. Moreover, we avoided technical language and terms. The booklet was developed for parents. However, illustrations may be understandable for children and adolescents as well.

The draft of the booklet covers 40 pages in total and provides written information with several elements supporting the content. Main topics of the booklet are: (1) communication about cancer and emotions within the family and with minors (10 pages), (2) living with cancer within the family and implementing a “new normal” family life (three pages), (3) reactions of children confronted with parental cancer (illustrated according to their developmental/age stages: infant, toddler, preschooler, school-aged child, and adolescent; three pages), and (4) dealing with disease progress and loss due to cancer (four pages). Additionally, the booklet includes addresses, contact details and a short description of local counseling services (100 km radius) and relevant homepages (10 pages), as well as literature suggestions for families (four pages). The booklet can be obtained by the corresponding author (LI).

### Participants

Affected parents or their partners were approached through cooperating oncological clinic wards at the University Cancer Center Hamburg and *via* the local psycho-oncological outpatient clinic at the University Medical Center Hamburg-Eppendorf. Parents were included when they met the following criteria: consent to participate, parenting at least one minor child (<18 years), and oncological diagnosis in one parent. Excluding criteria were the refusal of participation, insufficient German language skills, and self-rated cognitive deficiencies and bad health conditions (participants stated whether they were in the condition to participate or not). Experts who are working in the field of psychosocial care with families affected by parental cancer were contacted through a network address-list of institutions and experts working in the field of psychosocial support for families with parental cancer listed at the German Association of Psychosocial Oncology *via* mail or phone. Refusal of participation and insufficient knowledge of German were the only excluding criteria for experts.

### Qualitative Interviews

Interviews were conducted from August 2019 to October 2019 by phone or in person at the University Medical Center Hamburg-Eppendorf by the first author (LM, psychologist) using previously developed semi-structured interview guidelines (one for parents, one for experts). Interview guidelines were developed based on the research team’s professional experience and relevant literature as Helfferich (2009) and Kruse (2009). The content of the interview guidelines for experts and parents are displayed in Table 1. The first interviews were pilot interviews with one parent and one expert, conducted by LM and reviewed by the study team, and supported the suitability of the interview guidelines for experts and affected parents. As only minor changes were made, the pilot interviews were included in the analysis. At the beginning of each interview background information on the study, data protection, and organizational matters were introduced by the interviewer. All interviews were audio-recorded, transcribed verbatim, and the interviewer simultaneously took field notes. Participants could address open questions and make additional remarks at the end of every interview. In total, the collected data consist of 14 interviews including five expert interviews, five interviews with cancer patients, and four interviews with partners of cancer patients.

**Table 1:** Content of semi-structured interview guidelines with experts and affected parents.

Parents (patients and patient-partners)	Experts
(A) Demographic and disease-specific data	(A) Demographic and occupational data
(B) Experiences of cancer and parenting minor children (e.g., experiences and challenges)	(B) Parenthood and utilization of psychosocial support (e.g., need for psychosocial support)
(C) Information needs regarding cancer and parenthood (e.g., general need for information, specific information needs, information situation, and access to information)	(C) Information needs of cancer patients with minor children (e.g., general need for information, specific information needs, information situation of affected parents, and access to information)
(D) Evaluation of the developed information booklet as support offer (e.g., evaluation of content, scope and presentation, fulfillment of information needs, content comprehensibility, overview and structure, behavioral influence, optimal distribution time and path, and suggestions for improvement)	(D) Evaluation of the developed information booklet as a support offer for affected parents (e.g., evaluation of content, scope and presentation, perceived fulfillment of information needs, content comprehensibility, behavioral influence, optimal distribution time and path, and suggestions for improvement)
(E) Feedback on acceptance and usability of information booklet	(E) Feedback on acceptance and usability of information booklets in general
(F) Conclusion (additional remarks, questions)	(F) Conclusion (additional remarks, questions)

### Qualitative Analysis

The 14 audio datasets of the interviews were transcribed verbatim using f4-transcript software V.4.2 (Dresing and Pehl, 2015) and were analyzed using the structured content analysis following the approach of Mayring (2015). Interviews were analyzed separately for parents and experts. Still, coding guidelines (incl. themes and categories) marginally differed. Categories were generated deductively based on the guideline and theoretical considerations. Further categories, themes and subthemes were generated inductively based on the content of transcripts. A codebook including categories, themes and subthemes, and relevant anchor samples was developed by LM and LI. After finalizing the codebook, it was applied to all interview transcripts using the qualitative data analysis software MAXQDA (VERBI Software, 2019) by LM. LI and WG were involved in the coding process in case of unclear assignments of codes to the themes and subthemes. As the last step of data analysis, the coded text segments were summarized within the defined coding system. Quotes were translated from German to English by LM and LI. Sociodemographic variables were analyzed using descriptive statistics.

## Results

### Sample

Two cancer patients dropped out after receiving the booklet due to a bad health condition or insufficient language skills. Interviewed parents (patients and partners; *n* = 9) had between one and three children with 13 of 16 children being biological minors. Parents were between 38 and 54 years of age. All but one cancer patient and one partner were female. Four patients were not able to work due to their health condition and two partners also stated to be enrolled sick at work. The majority of families (*n* = 7) received parental cancer diagnosis within the last 10 months prior to the interview. All patients underwent chemotherapy. Other treatments among the patients were surgery, radiotherapy, and medication. All interviewed parents were in committed relationships and lived with their partners and children in one household. Interviews with parents ranged between 26 and 67 min with an average of 46 min.

All interviewed experts (*n* = 5) were female with an age range from 34 to 46 years. Their work experience included psycho-oncology, family counseling in the field of parental cancer, psychotherapy (adults or children), and educational psychology. Expert’s work setting varied from counseling services for cancer patients with minor children to consultation hours in a clinical setting. Interviewed experts had a work experience in the field of parental cancer between 5.5 and 13 years. Interviews with experts took on average 58 min (range: 46–93 min).

### Feedback on Acceptability and Usability of the Information Booklet

Participants provided feedback on the first draft of the developed information booklet. Acceptability and usability of the booklet was assessed using the feedback and qualitative evaluation of the study participants on the structure, presentation, understandability, and content of the booklet.

#### Structure, Presentation, and Understandability

Overall, experts and parents evaluated the length and the structure of the booklet as adequate, e.g., it was mentioned that it was “short” and “quick to read” with the relevant aspects to be addressed. Therefore, it would allow affected parents to gain relevant knowledge “without exhausting” themselves and would answer parents’ main questions “without getting lost in detail.” The structure was evaluated as following the “natural order of events,” which would make the booklet “self-explanatory” and “time-saving.” Moreover, the information booklet was rated as “easy to understand and to read.” Most participants stated that the illustrations and text boxes underlined the content of the text and supported the readability. The presentation and illustration of information was perceived appealing to parents with minor children by experts and parents. Some participants evaluated single illustrations as inappropriate, e.g., too sad. Some parents reported that they looked at the booklet together with their children.

#### Content

Regarding the content, the booklet was mentioned to be “very helpful,” to cover the “most important topics,” and one participant highlighted the “ideas, practical tips, and suggested phrases.” According to most experts, the content of the booklet was perceived to be congruent to the information given in counseling or by psychosocial support institutions. Most participants evaluated the included topic on disease progress, farewell and death as necessary and important. The presentation of age-specific information was appreciated. Interviewed parents stated to be grateful for the “guidance” on how to deal with the situation. It was rated as helpful, that the recommendations were written down in “black and white” and could always be looked up again. It was mentioned, that having permanent access to professional information could help regarding the “information policy” for children. Especially, the structured information regarding support offers and contact data of local counseling centers were reported to be urgently needed and helpful.

### Information Needs

Since themes and categories only marginally differed between interviewed experts and parents, combined results are presented.

Experts and parents reported the existence of information needs regarding parental cancer. The following dimensions of information needs were identified: (1) communication within the family, (2) support offers, (3) children’s disease understanding and needs, (4) organization of family life, (5) competence in parenting, and (6) additional information material. Table 2 shows a list of specific information needs within these dimensions.

**Table 2:** Specific information needs of affected parents regarding parental cancer reported by study participants (*n* = 5 patients, *n* = 4 partners, and *n* = 5 experts).

**(1) Communication with children** Information about cancer Information depth Wording and terminology Age-appropriateness (cognitive understanding of diagnosis) Responses to “shocking” questions Survival rates and uncertainties Side effects (e.g., hair loss) Farewell and death	Timing of communication Initial diagnosis Occurrence of reduced parental resources and needs Occurrence of side effects Cancer recurrence Disease progress and death Ways of communication Setting Channel and supporting media Inclusion of others (e.g., teachers) Withdrawal and refusal of child (minor refuses communication)
**(2) Support offers** Contact details Description and presentation of support offers Financing Age-appropriate support offers/options for children	Eligibility criteria/target group Utilization process Obligations and time investment
**(3) Children’s understanding and needs** Assessment of minor’s wellbeing Minor’s resilience and potential reactions (developmental, atypical)	Handling impacts of treatment on interactions and attentional deficits Dealing with emotions (e.g., fear, anger) Options for inclusion of children
**(4) Organization of family life** Household and chores reorganization Organization of childcare and support	Hospital visits of minors Separated/Single parents: e.g., accommodation of child, child custody
**(5) Competence in parenting** Reassurance of own behavior and choices (strengthening parental self-efficacy)	Dealing with changes in the parental role Overcoming feeling of helplessness
**(6) Additional information material** High quality and eligible sources of further information (e.g., websites)	Sources of age-specific information for children affected by parental cancer

According to most interviewees, there is a need for information on communication about cancer diagnoses and related aspects within the family. Central aspects are questions on the timing, the content and the way of communication (e.g., what is the right wording talking to children?). Furthermore, many experts and parents reported the need for information on specific support offers for affected families. Information needs regarding the possible impact and consequences of parental cancer on the child (e.g., risk of developing psychological problems) and on the reorganization of family life were mentioned. Another information need refers to the cognitive understanding of cancer and age-specific ways of coping. Many parents reported their need of reassurance and information on questions such as “*Can I do anything wrong*?” or “*Am I still a good parent*?”

According to the experts, there might be specific information needs that should be taken adequately into account for divorced parents, single parents, parents with very young children, and parents struggling with the situation of disease themselves. It was reported that certain situations might be leading to increased levels of information needs (e.g., shortly after diagnosis, disease progression or recurrence, and palliative situations). Furthermore, the time prior to the diagnosis while waiting for test results was reported to be stressful for the parents and leading to unmet information needs regarding the involvement of their children during this time. Other situations with high levels of information needs mentioned by the experts were social situations (e.g., minor’s school enrolment), previous traumatic experiences with cancer, low levels of social or childcare support as well as child’s behavioral difficulties or puberty.

As preferred sources for information, the participants mentioned medical consultations, internet resources as well as booklets. While the internet was perceived as least helpful and satisfying, the combination of a booklet and a personal contact with a physician, psychologist, or psycho-oncologist was identified as an important source of information. Patients reported that the access to information was perceived as insufficient with difficulties to find suitable information for their situation. Additionally, experts reported a lack of adequate supporting materials and the need for an improved integration of information transfer into clinical routines.

### Suggestions for Implementation

Several factors that could influence the realization of recommendations in the booklet were mentioned: (1) the way the booklet was handed over, (2) the educational level of the parents, and (3) the provision of additional professional support, if needed. A personal handing out of the booklet with verbal instructions was suggested to enhance the positive effect of the booklet. If a personal handout would not be feasible, it was suggested to be helpful to display and offer the booklet in hospitals and practices. Both, experts and parents suggested to hand out the booklet in an early phase of the cancer journey (e.g., with diagnosis). Moreover, it was suggested that, where necessary, an accompanying personal counseling could present a complementary individualized approach for affected families.

### Adaptation of the Information Booklet Based on Collected Data

As both, experts and participants reported that the information booklet fulfills the main information needs of affected parents, only minor changes were made in the developed information booklet.

#### Suggestions for Improvement

Besides suggestions for single phrases and sentences or presentation of the content, participants suggested additions with regard to the content. According to the participants, some aspects could be addressed more extensively, e.g., “household help,” “health insurance,” “sick pay,” “involvement of educators,” “mutual activities during strong parental impairment,” “risk of infection,” and “protective measures.” Both experts and parents claimed that the advice to openly talk with the children about the parental cancer or to get psychosocial counseling could have been more direct in the booklet. Experts as well as parents reported that it is crucial to help parents recognize the necessity of talking and involving their children regarding the parental cancer. It was also recommended that specific information needs of divorced parents and single parents are directly addressed in the booklet. Participants suggested it would be helpful to offer the booklet in different languages.

#### Incorporated Changes

In the revision, aspects on the involvement of school personnel or daycare as well as aspects on the risk of infection were extended. We refrained from a more comprehensive presentation of topics concerning social law (e.g., health insurance, household help) due to length issues, but added contact information for counseling centers. More text boxes to highlight important aspects were added to make it easier to scan the booklet for the different themes and provide quick and brief information. A section highlighting the importance that parents also need to care for themselves to make sure they are emotionally available for their children as an encouragement to not only seek help for their children but also for themselves with contacts for psycho-oncological support offers was also included. The illustrations, though occasionally rated as too sad, were not altered. The illustrator aimed at neutral facial expressions to make sure the situation is neither marked as too gloomy nor as too cheerful.

## Discussion

The findings of the study indicate the usability of our information booklet for parents affected by cancer. Overall, the structure, presentation, and understandability as well as the content were evaluated as helpful and supportive by experts and affected parents. Still, few recommendations for improvement were mentioned.

The reported information needs on communication within the family, children’s disease understanding and needs, and organization of family life are concurrent with a recent study on needs (Schiena et al., 2019). Those needs were mainly met within the information booklet. However, for the implementation of the booklet, some interviewed experts and parents suggested that a combination of the booklet and a personal contact with a physician, psychologist, or psycho-oncologist might serve as a promising source of information. Parents need easy access to information without extensive searching for information on the internet and evaluating the quality of information they find by themselves (Schiena et al., 2019). This supports a stepped-care model approach including the developed information booklet. Besides the easy and fast access to information, the information booklet could also possibly lead to positive dynamics within the family due to incorporated practical suggestions. A recent study by Konings et al. (2020) about the development of a booklet for parents affected by cancer with adolescent and young adult children in English language had concurrent findings regarding most critical information needs (importance and handling of communication about cancer with children) as well as the anticipated and experienced positive effects of the developed information booklet (confidence as parents and in communicating with their children).

### Study Limitations

This study provides results on a qualitative evaluation of a newly developed information booklet. The sample was small and rather homogenous regarding cultural, educational, family, and economical background. Hence, the composition of the subsamples (experts, parents) does not allow generalizability and conclusions are not universally applicable. Moreover, vulnerable groups such as single parents or highly burdened parents without access to psychosocial help as well as men are underrepresented in this study. Many participants already had received some kind of psychosocial support limiting the generalizability of the results. Parents as well as experts were interviewed after receiving and reading the booklet. Hence, findings on the information needs may be biased by the information provided in the booklet. Still, parents and experts mentioned new aspects or emphasized certain needs apart from the presented booklet. Additionally, coding by one coder might impact the reliability and social desirability biases could have influenced the results.

### Clinical Implications

Besides information provision of healthcare professionals, booklets and internet resources represent the main sources for information. Patient-centered information congruent to information needs is highly relevant and is associated with patient satisfaction and quality of life (Husson et al., 2011). Hence, information provision should be included into routine care as a basic intervention. Providing detailed information, e.g., on contact details for support offers may reduce barriers for the use of psychosocial support services. The developed information booklet can be a promising approach to provide a basic support for families affected by parental cancer as part of a stepped-care model and it was reported to provide a helpful source of information. We revised the information booklet after the pilot evaluation. Nonetheless, the need for practical and financial support of affected families could only partially be addressed, e.g., due to complex social legal aspects. However, contact details for further questions in this aspect were added. A further step would be the provision of the booklet in other languages.

According to the results from this qualitative study, best way and timing for the distribution of the booklet may be when receiving the diagnosis, respectively in an early phase of the illness. Moreover, a personal handout with verbal instructions by a healthcare professional was suggested.

## Conclusion

The results of this qualitative study suggest a high level of acceptance and good usability of the developed booklet for affected parents. Specific information needs were identified for different dimensions, which were generally covered by the information booklet illustrating the necessity to provide information to cancer patients parenting minor children according to their specific needs. The information booklet was revised based on the assessed data and few information were added. Based on our findings, the revised information booklet can be helpful by providing necessary information for affected families. Moreover, the booklet can serve as a low threshold intervention and therefore might facilitate the access to psychosocial and psycho-oncological care. Effects of the booklet, e.g., on the actual use of psychosocial support services and outcome parameters such as parental self-efficacy and psychological distress should be investigated in a larger study.

## Data Availability Statement

The datasets presented in this article are not readily available because the datasets for this study cannot be shared publicly due to the sensitive and personal nature of the interview transcripts. Because of ethical reasons and potentially identifying patient information, data (in German language only) are available upon reasonable request after consultation of the responsible ethics committee and data protection office. Requests to access the datasets should be directed to l.inhestern@uke.de.

## Ethics Statement

The studies involving human participants were reviewed and approved by the Ethics Committee of the Chamber of Psychotherapists of Hamburg. The patients/participants provided their written informed consent to participate in this study.

## Author Contributions

LM, WG, and LI developed and designed the study. LM and LI wrote the first draft of the manuscript. LM conducted the interviews and performed the data analysis. All authors discussed the results and contributed to the final manuscript.

## Conflict of Interest

The authors declare that the research was conducted in the absence of any commercial or financial relationships that could be construed as a potential conflict of interest.

## Publisher’s Note

All claims expressed in this article are solely those of the authors and do not necessarily represent those of their affiliated organizations, or those of the publisher, the editors and the reviewers. Any product that may be evaluated in this article, or claim that may be made by its manufacturer, is not guaranteed or endorsed by the publisher.
